# Supplementary material for: Urinary Exosomal Cystatin C and Lipopolysaccharide Binding Protein as Biomarkers for Antibody−Mediated Rejection after Kidney Transplantation
Source: Biomedicines. 2022 Sep 21;10(10):2346. doi: 10.3390/biomedicines10102346 (PMC9598834; doi:10.3390/biomedicines10102346)
Supplement: Supplementary file 1 [file biomedicines-10-02346-s001.zip › biomedicines-1788686-supplementary.pdf]

Supplementary Materials:

Supplementary Methods

Western blot analysis for the validation of ABMR-specific urinary exosomal proteins

Exosomal proteins were lysed using radioimmunoprecipitation assay buffer containing protease inhibitor cocktails. Protein samples (10 μg) were resolved using polyacrylamide gel electrophoresis and transferred into a polyvinylidene difluoride membrane. The membrane was probed with primary antibodies against each protein for 1 h at room temperature, and incubated with horseradish peroxidase-linked secondary antibody for 1 h at room temperature. The blots were developed using an enhanced chemiluminescence detection reagent (Bio-Rad Laboratories, Hercules, CA, USA) and exposed using a Bio-Rad Gel Documentation System (Bio-Rad). Image J Software (NIH, Bethesda, MD, USA) was used for densitometry analysis.

Immunohistochemistry (IHC)

Formalin-fixed, paraffin-embedded tissue sections were immunohistochemically stained using the OptiView DAB IHC Detection Kit on a BenchMark XT automatic immunostaining device (Ventana Medical Systems, Tucson, AZ, USA) according to the manufacturer's instructions. Four-micrometer-thick sections obtained with a microtome were transferred onto silanized charged slides and allowed to dry for 10 min at room temperature, followed by incubation for 20 min at 65°C. Sections were processed by heat-induced epitope retrieval method using Cell Conditioning 1 buffer for 32 min and incubated for 16 min with antibodies in an autoimmunostainer. Antigen-antibody reactions were visualized using the Ventana OptiView DAB IHC Detection Kit (Optiview HQ Linker 8 min, Optiview HRP Multimer 8 min, Optiview H2O2/DAB 8 min, Optiview Copper 4 min). Counterstaining was performed using Ventana Hematoxylin II for 12 min and Ventana Bluing reagent for 4 minutes. Finally, all slides were removed from the stainer, dehydrated, and cover-slipped for microscopic examination.

Supplementary Figures

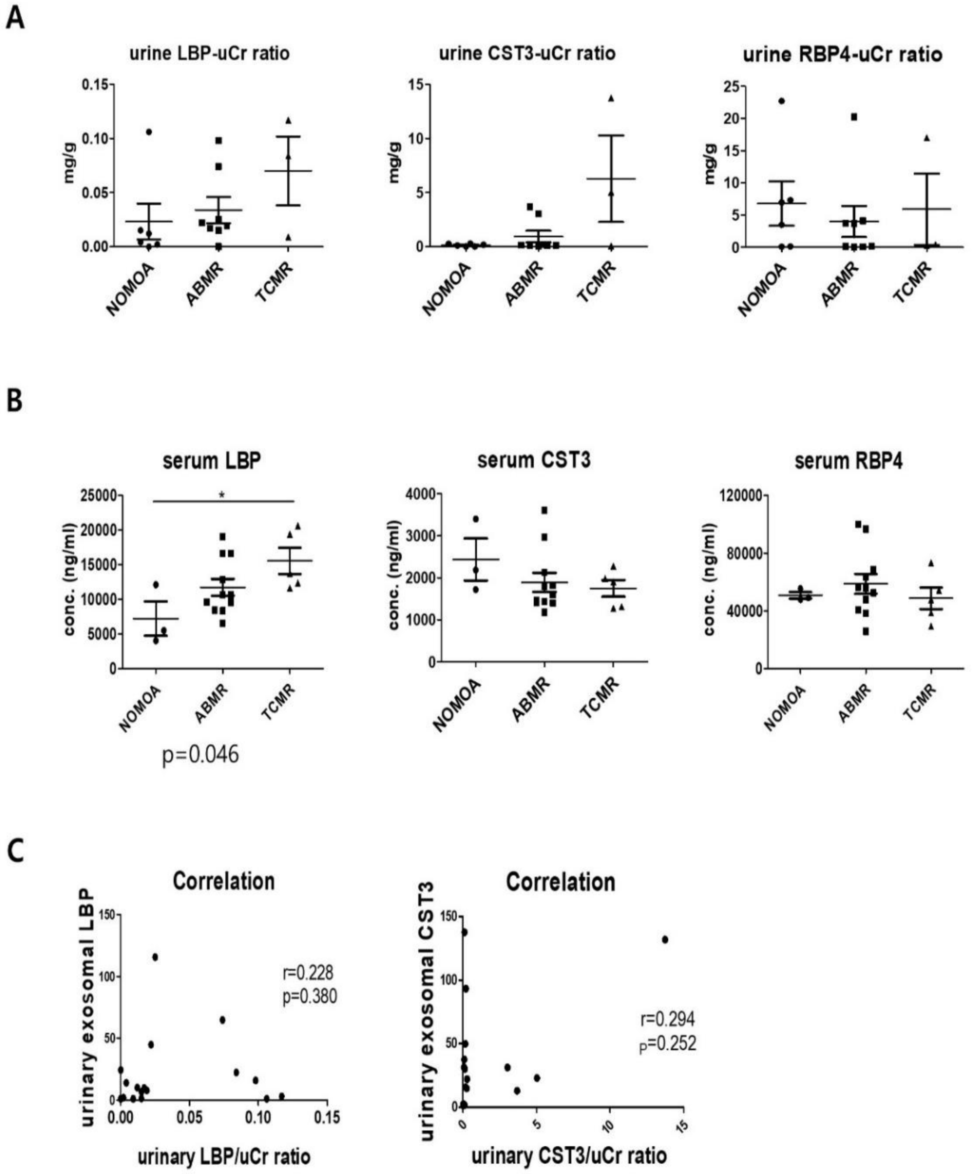

Figure S1. Validation of proteomic biomarkers in urine and serum. A. The creatinine (Cr) levels of urine and serum were evaluated from matched patients in WB-based validation (urine; 6 NOMOA, 8 ABMR, and 3 TCMR, serum; 6 NOMOA, 8 ABMR, and 4 TCMR). B. The protein concentration in urine was measured by ELISA and adjusted to urine creatinine level (9 NOMOA, 10 ABMR, and 4 TCMR). C. The protein concentration in serum was measured by ELISA (3 NOMOA, 11 ABMR, and 5 TCMR). Error bars indicate mean +/− SEM of individuals included in each group. Kruskal-Wallis One-way ANOVA p values are indicated for each protein. Dunn's Multiple Comparison test post hoc test significance values are indicated as **P*< 0.05, ***P*< 0.01, and ****P*< 0.001. D. Analysis of correlation between urinary exosomal proteins and urinary free proteins was assessed using Spearman's rank correlation (n=17). The r of 0.2-0.29 in Spearman's is considered to have a weak correlation. ABMR, antibody-mediated rejection; NOMOA, no major abnormality; TCMR, T cell-mediated rejection; CST3, Cystatin-C; LBP, lipopolysaccharide-binding protein; uCr. urine creatinine.

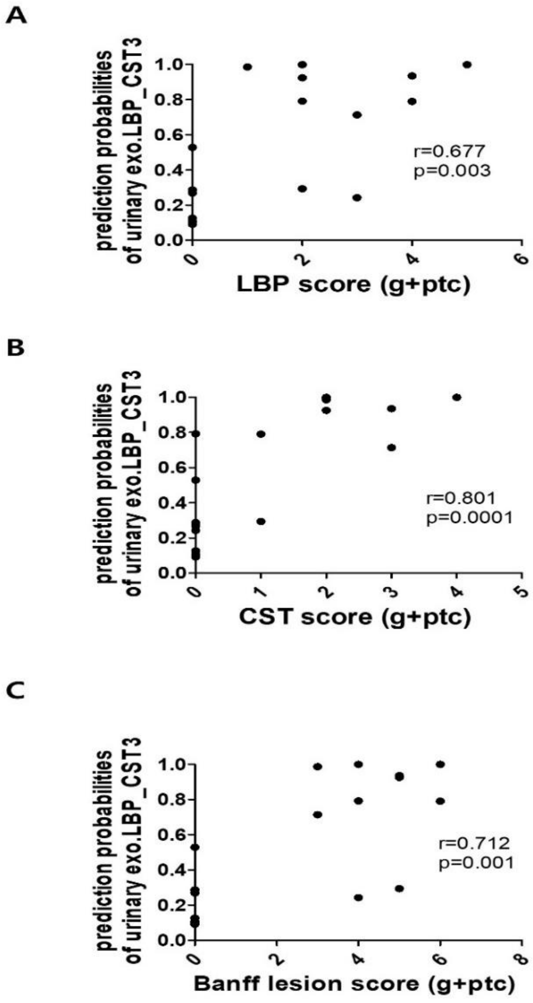

Figure S2. Association of prediction probabilities of combined urinary biomarkers with expression in allografts. Correlation between prediction probabilities of combined urinary biomarkers with (A) LBP score, (B) CST3 score, and (C) Banff lesion score (g+ptc) from matched samples (n= 17) are shown. The results were categorized as strong (r=0.7–1) and moderate (r=0.5–0.7) according to the degree of association. ABMR, antibody-mediated rejection; TCMR, T cell-mediated rejection; CST3, Cystatin-C; LBP, lipopolysaccharide-binding protein; g, glomeruli; ptc, peritubular capillaries.

Supplementary Tables

Table S1. Comparison of histologic findings of for-cause biopsies of each group.

	**Discovery cohort**					**Validation cohort**			
	**ABMR (N=12)**	**BKVN (N=5)**	**TCMR (N=8)**	**NOMOA (N=10)**	**P**	**ABMR (N=25)**	TCMR (N=10)	NOMOA (N=19)	P
Banff score									
g	2.00±1.04	0.20±0.45	0.38±0.45	0.33±0.50	<0.001	1.96±0.84	0.20±0.42	0.33±0.49	<0.001
ptc	2.33±0.78	0.80±0.84	0.50±1.07	0.11±0.33	<0.001	2.40±0.65	0.40±0.70	0.16±0.50	<0.001
t	0.50±0.91	1.20±1.10	2.38±0.52	0.11±0.33	<0.001	1.04±0.20	2.50±0.71	0.21±0.42	<0.001
i	1.00±0.85	1.40±1.14	2.25±0.46	0.44±0.53	0.001	1.40±0.65	2.10±0.57	0.42±0.51	<0.001
cg	1.67±1.16	0	0	0.67±0.71	<0.001	1.32±1.11	0.20±0.42	0.28±0.75	<0.001
ci	2.08±1.00	1.40±1.34	1.13±0.84	1.56±1.13	0.223	1.88±0.93	1.20±0.79	1.16±0.96	0.027
ct	2.17±0.84	1.20±1.10	1.25±0.71	1.56±1.13	0.12	1.92±0.86	1.40±0.70	1.21±0.92	0.032
cv	1.33±0.89	1.00±0.71	1.13±0.99	1.44±1.13	0.846	0.92±0.98	1.50±0.97	1.11±0.94	0.28
v	0.17±0.39	0	0.63±0.92	0	0.114	0	0.20±0.63	0.05±0.23	0.344
ti	2.00±0.85	2.20±1.30	2.50±0.54	1.33±0.87	0.063	1.84±0.85	2.20±0.79	1.0±0.67	0.001
C4d > 0 on IHC	5 (41.7)	1 (20)	1 (12.5)	1 (11.1)	0.083	12(48.0)	3(30.0)	3(15.8)	0.229

Values are mean ± standard deviation or n (%).
ABMR, antibody-mediated rejection; BKVN, BK virus nephropathy; TCMR, T cell-mediated rejection; NOMOA, no major abnormality; BMI, body mass index; ESRD, end-stage renal disease; FSGS, focal segmental glomerulosclerosis; PCKD, polycystic kidney disease; KT, kidney transplantation; ATG, anti-thymocyte globulin; HLA, human leukocyte antigen; DDKT, deceased donor kidney transplantation.

Table S2. List of eighteen urinary exosomal biomarker candidates.

Accession	Gene name	Description	Peptides	Unique Peptides	Abundance Ratio: (ABMR/NOMOA)	Fold change [ABMR /NOMOA (log²)]	P-Value
**P20062**	**TCN2**	**Transcobalamin-2**	**3**	**3**	4.436	2.149259365	0.00095445
P02652	APOA2	Apolipoprotein A-II	3	3	4.085	2.030336078	9.45E-07
Q9UGM5	FETUB	Fetuin-B	7	7	4.342	2.118359726	1.28E-08
O14896	IRF6	Interferon regulatory factor 6	4	4	4.395	2.135863165	0.00045703
P05546	SERPIND1	Heparin cofactor 2	19	19	3.667	1.874600266	7.34E-06
Q99972	MYOC	Myocilin	11	11	5.61	2.488000771	6.48E-09
P06681	C2	Complement C2	28	28	3.37	1.752748591	2.43E-05
P26572	MGAT1	Alpha-1,3-mannosyl-glycoprotein 2-beta -N-acetylglucosaminyltransferase	8	8	3.104	1.634128558	6.07E-06
P18428	LBP	Lipopolysaccharide-binding protein	15	15	3.655	1.869871406	7.69E-06
P27169	PON1	Serum paraoxonase/arylesterase 1	13	13	4.935	2.303050085	6.45E-08
P08697	SERPINF2	Alpha-2-antiplasmin	14	14	3.009	1.589284107	0.00010906
P00734	F2	Prothrombin	36	36	3.597	1.846794159	9.72E-06
P02647	APOA1	Apolipoprotein A-I	23	23	3.143	1.652142272	6.21E-05
P29622	SERPINA4	Kallistatin	22	22	3.368	1.751892138	2.45E-05
P15291	B4GALT1	Beta-1,4-galactosyltransferase 1	12	12	3.045	1.606442228	9.35E-05
P01034	CST3	Cystatin-C	6	6	4.863	2.281846592	1.24E-08
P01860	IGHG3	Immunoglobulin heavy constant gamma 3	13	5	4.256	2.089498151	7.56E-07
P02753	RBP4	Retinol-binding protein 4	11	11	3.902	1.964213778	2.92E-06

Table S3. Area under the curve of the receiver operating characteristics curves of the four candidate biomarkers in differentiating AMBR from NOMOA in the validation cohort.

	AUC	95% CI	p-value	Cut-off	Specificity	Sensitivity	PPV	NPV
**LBP**	0.813	0.562–0.954	0.0094	>6.04	62.5	100	76.9	100
**CST3**	0.802	0.555–0.951	0.0068	>22.06	100	70	100	72.7
**PON1**	0.619	0.349–0.844	0.4008	>5.130	85.71	55.56	83.3	60
**RBP4**	0.532	0.273–0.779	0.8418	<8.403	57.14	66.67	66.7	57.1
**LBP-CST3**	0.888	0.651–0.986	<0.0001	>0.529	80	87.5	81.8	85.7

ABMR, antibody-mediated rejection; NOMOA, no major abnormality; CI, confidential interval; PPV, positive predictive value; NPV, negative predictive value; LBP, lipopolysaccharide-binding protein; CST3, Cystatin-C; PON1, serum paraoxonase/arylesterase 1; RBP4, retinol-binding protein 4.

Table S4. List of antibodies used for Western blot analysis and Immunohistochemistry.

Antibody	Host	Clonality	Company	Dilution factor	Application
CFD	mouse	monoclonal	Invitrogen	1:200	Western blot analysis
PON1	mouse	monoclonal	Abcam	1:200	Western blot analysis
CST3	goat	polyclonal	R&D systems	1:500	Western blot analysis
RBP4	goat	polyclonal	R&D systems	1:2000	Western blot analysis
LBP	goat	polyclonal	R&D systems	1:2000	Western blot analysis
CD63	mouse	monoclonal	BD Biosciences	1:1000	Western blot analysis
TSG101	mouse	monoclonal	GeneTex	1:1000	Western blot analysis
Alix	mouse	monoclonal	Cell signaling Technology	1:1000	Western blot analysis
CST3	goat	polyclonal	R&D systems	1:100	Immunohistochemistry
RBP4	goat	polyclonal	Affinity Biosciences	1:500	Immunohistochemistry
LBP	goat	polyclonal	Invitrogen	1:300	Immunohistochemistry
